# Detection of butane and propane gases via C_2_N sensors: first principles modeling

**DOI:** 10.1038/s41598-023-46870-x

**Published:** 2023-11-07

**Authors:** Asma Wasfi, Mawahib Sulieman, Ziad Sefelnasr, Abdulla Alteneiji, Atawulrahman Shafiqurrahman, Ammar Alharairi, Falah Awwad

**Affiliations:** 1 Department of Electrical and Communication Engineering, College of Engineering, United Arab Emirates University, P. O. Box 15551, Al Ain, United Arab Emirates; 2 College of Engineering, Al Ain University, Al Ain, United Arab Emirates

**Keywords:** Natural hazards, Engineering, Materials science, Nanoscience and technology

## Abstract

Gas sensing is a critical research area in aerospace, military, medical, and industrial environments, as it helps prevent risks to human health and the environment caused by toxic gases. Propane and butane, commonly used as fuels in household and industrial settings, are toxic and flammable gases that need to be effectively detected to avoid leakage or explosion accidents. To address this, nanomaterial-based gas sensors are being developed with low power consumption and operating temperatures. In this study, two-dimensional nitrogenated holey graphene (C_2_N) based sensors are used for the first time for the identification of butane and propane gases. The sensor consists of two C_2_N electrodes connected via a C_2_N channel. The C_2_N sensor design was enhanced by replacing the C_2_N electrodes with gold electrodes and adding a gate terminal under the channel. The resistive method is employed to detect butane and propane gases by measuring the variation in the electrical conductivity of the sensor due to exposure to these target molecules. To investigate the electronic transport properties, such as transmission spectra, density of states and current, first principles simulations of the C_2_N-based sensors is conducted using Quantumwise Atomistix Toolkit (ATK). The detection method relies on the alteration of the FET's electrical current at specific gate voltages due to the presence of these gases. This proposed sensor offers the potential for small size and low-cost gas sensing applications. The designed sensor aims to effectively detect propane and butane gases. By leveraging the unique properties of C_2_N and utilizing advanced simulation tools, this sensor could provide high sensitivity and accuracy in detecting propane and butane gases. Such an advancement in gas sensing technology holds significant promise for ensuring safety in various environments.

## Introduction

Gas sensing has attracted significant importance across various areas. The detection of toxic gases is critical to prevent accidents in both household and industrial environments. Therefore, the development of effective tools for detecting the presence of these gases is highly important^1–7^. Achieving a high level of sensitivity and resolution is the ultimate objective in gas detection. Detecting the presence of desired gases, even at very low concentrations, is crucial. Despite the use of solid-state gas sensors, achieving such high resolution has remained challenging^2, 3, 8^. The primary cause of the low performance in these devices is attributed to sudden fluctuations and defects caused by the charge carriers thermal motion, resulting in the generation of noise within the device^9^.

Solid-state gas detectors can be classified into various categories based on their working principles. The most prevalent categories include resistive type sensors, impedance type gas sensors (utilizing alternating current measurements), electrolyte-based gas sensors, and semiconductor gas sensors. Among these, resistive solid-state gas detectors are the most commonly used due to their low fabrication cost and simplicity. These devices operate by detecting changes in the semiconductor material resistance due to its interaction with the target gas. The alteration in resistance is attributed to the movement of charge carriers among the semiconductor material and the target gas^10, 11^. Impedance-based gas devices exhibit a change in the device frequency response upon exposure to the required gas molecules^12^. Conversely, solid-state electrolyte-based gas detectors rely on alterations in the electrolyte ionic conductivity, which occur due to the movement of charge carriers (electrons or holes) from the targeted gas molecules^13^. Solid-state gas sensors play a critical role in monitoring and controlling the release of toxic and hazardous gases. However, these sensors do possess certain limitations concerning selectivity, sensitivity, reproducibility, and long-term stability. Despite the increasing demand for gas sensors, there remains a requirement to design sensors that operate at low temperature, highly sensitive, robust, and reversible. Nanomaterials have emerged as potential candidates for developing gas sensors that operate at low temperatures and consume less power. It has been observed that nanomaterials can identify a wide range of inorganic and organic molecules. The gas sensing material sensitivity is primarily determined by its surface to volume ratio, which is significantly high for nanomaterials. Nanomaterials have the advantage of a high surface-to-volume ratio, allowing them to efficiently adsorb detectable target molecules^14^. Due to their extraordinary electronic properties, graphene has emerged as an intriguing and promising substitute for conventional solid-state gas sensors. Graphene and its derivatives exhibit exceptional characteristics that make them well-suited to replace traditional gas sensors^15–17^.

Graphene-based field-effect transistors (FETs) show promise as potential candidates for detecting a wide range of gases, biomolecules, toxic compounds, offering improved sensitivity in comparison to solid state sensors^18–21^. The range of sensitivity for graphene-based FETs typically spans from parts per million (ppm) to parts per billion (ppb)^11, 18, 22^. In FET-based gas sensors, the flow of electric current across the sensor is controlled by the gate electrode. The presence of target molecules affects the charge carriers concentration within the graphene membrane, resulting in a change in the current passing across the sensor at a specific gate voltage. Certain gas molecules act as charge carrier acceptors for graphene, reducing the electric current after adsorption, while others act as donors, increasing the current. This alteration in the current reading serves as a detection signal for the target molecules^23, 24^. Moreover, conductance fluctuation can also be utilized as a detection signal^25^.

Nitrogen-doped carbon nanomaterials exhibit superior performance in biosensors compared to pristine carbon, making them highly suitable for such applications^26^. The utilization of nitrogen-doped carbon nanomaterials in biosensors is driven by their unique characteristics. Notably, carbon nanomaterials, such as graphene and carbon nanotubes, possess high surface-to-volume ratios, enabling effective adsorption of numerous biomolecules onto their surfaces^27^. Introduction of nitrogen atoms into carbon nanomaterials enhances their conductance, thereby improving their performance and sensitivity^28^. The incorporation of nitrogen into carbon nanomaterials can be achieved through a doping process, resulting in materials that exhibit improved stability and enhanced electrical conductivity compared to pristine carbon. Nitrogen atoms act as electron acceptors, thereby improving the carbon nanomaterials electrical conductivity and enhancing the biosensing applications sensitivity^29^. Moreover, nitrogen doping improves the chemical stability of carbon nanomaterials, making them more resistant to degradation. Nitrogen-doped carbon nanomaterials demonstrate reduced toxicity and enhanced stability in biological environments, thus increasing their biocompatibility. Furthermore, in contrast to pristine carbon, nitrogen-doped carbon nanomaterials have exhibited superior stability and biocompatibility, which makes them exceptionally well-suited for biosensor applications^30^. In summary, nitrogen-doped carbon nanomaterials offer desirable features, including a high electrical conductivity, a large surface area, , and enhanced biocompatibility, making them excellent candidates for utilization in biosensors^26, 28, 30^.

Two-dimensional nitrogenated holey graphene (C_2_N) have gained significant prominence in the research field over the past decade. As silicon technology reaches its geometric limits, the need for alternatives that can replace it becomes crucial. The outstanding chemical, optical, electrical, and mechanical properties of two-dimensional nitrogenated holey graphene have positioned it as a promising contender for upcoming nanoelectronics applications^31, 32^. The utilization of two-dimensional nitrogenated holey graphene-based sensors has become increasingly important in a broad range of sensing applications, such as the detection of gas molecules, chemicals, and biomolecules^31, 33–37^. Its ease of use in creating electrical contacts and manipulating them based on specific requirements surpasses that of other nanomaterials.

The novelty of this study lies in the use of C_2_N material as a sensor for the identification of individual propane and butane gas molecules. To the best of our knowledge, this is the first research that employs C_2_N material to detect propane and butane molecules.

This work encompasses a comprehensive investigation into the development and characterization of a gas sensor utilizing C_2_N for the identification of butane and propane gases. The study begins by presenting the sensor setup and configuration, outlining the design modifications made to enhance its performance. A detailed description of the computational method employed, namely first principles simulations using Quantumwise Atomistix Toolkit (ATK), is provided to elucidate the electronic transport characteristics of the C_2_N-based sensors. The obtained results, including the density of states and current profiles, are thoroughly analyzed to gain insights into the gas detection mechanism and evaluate the sensor's potential sensitivity and accuracy. The article sheds light on the advancements achieved in gas sensing technology through the application of C_2_N-based sensors and highlights their promising prospects in small size and low-cost gas sensing applications. By using computational simulations, this work offers a comprehensive understanding of the detection capabilities of the proposed sensor and paves the way for further advancements in ensuring safety and mitigating risks associated with propane and butane gases in various environments.

## Materials and methods

This work was conducted using Quantumwise Atomistix Toolkit (QuantumATK 2018.06) and its graphical user interface (Virtual Nanolab). To perform the ATK-VNL simulations, the High Performance Computing (HPC) resources provided by the United Arab Emirates University were utilized. The HPC system involved the utilization of seven nodes, each equipped with 36 processors, resulting in a total of 252 processors being employed to successfully perform the simulation tasks.

### Sensor setup and configuration

**Figure 1:**
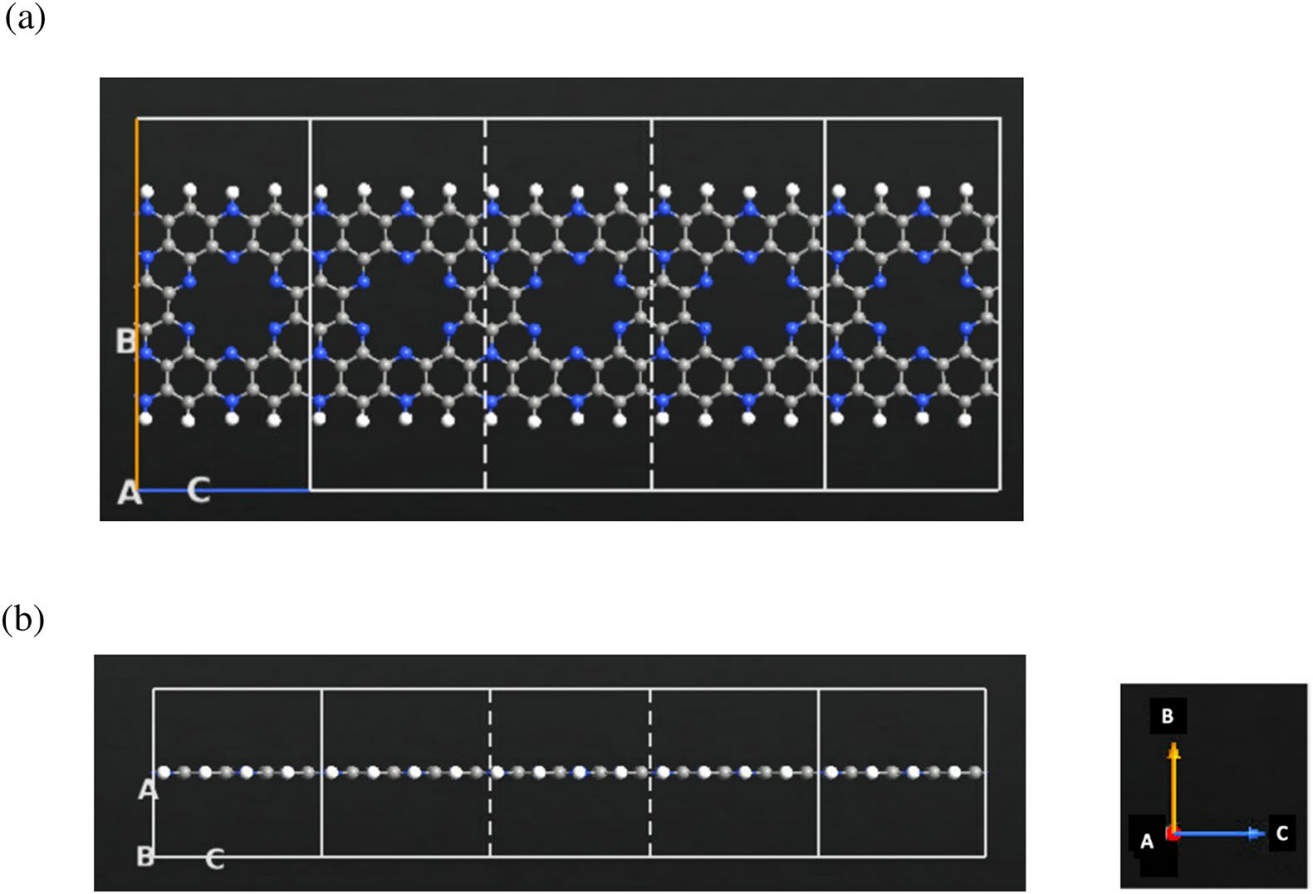
C_2_N based device built by QuantumATK. (**a**) Schematic view of a monolayer C2N sensor. (**b**) Cross sectional representation of the sensor. Color code: carbon-gray, nitrogen-blue, and hydrogen-white.

The configuration of the C_2_N-based device were thoroughly studied using QuantumATK software. Figure 1 illustrates the nanoscale sensor settings employed. The C_2_N system comprises C_2_N electrodes and a C_2_N central region consisting of a single layer of C_2_N, without the inclusion of a gate. The C_2_N channel exhibits a width of 13 Å and a length of 8 Å, while the length of the C_2_N electrodes is 8 Å. The entire system is made of 149 atoms. To detect the distinctive electronic signatures of each target molecule: propane and butane, first-principle electronic transport characteristics were performed. In Fig. 1, the indicators A, B, and C represent the A-, B-, and C-directions, respectively.

**Figure 2:**
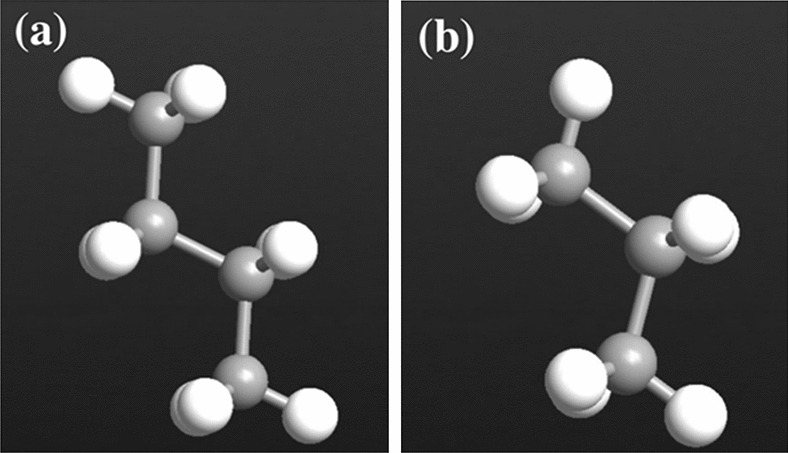
Atomic structure for each target molecule. (**a**) Butane. (**b**) Propane. Color code: hydrogen-white, and carbon-gray.

Figure 2 illustrates the atomic structure of the target molecules, (a) butane, and (b) propane gases, highlighting their unique chemical and electronic composition. Each target molecule exhibits a unique electronic current due to these characteristics.

**Figure 3:**
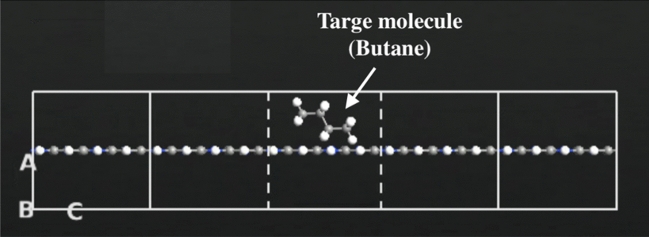
Schematic representation of the C_2_N sensor with Butane molecule.

In Fig. 3, the C_2_N sensor structures are depicted with the inclusion of butane molecule. The prominent hollow site within the channel displayed in Fig. 1 represents the most stable adsorption site for the target molecule, as it allows for the efficient adsorption of each gas molecule^31^. Throughout the analysis, a finite bias voltage ranging from 0 to 0.5 V was applied between the right and left electrodes.

**Figure 4:**
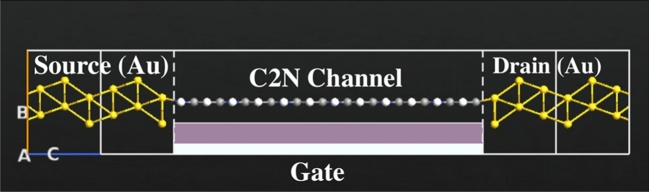
Schematic representation of the enhanced C_2_N sensor. The constructed sensor comprises of two gold electrodes, a single-layer of C_2_N to form the channel, and a gate terminal. Color code: gold-yellow, carbon-gray, nitrogen-blue, and hydrogen-white.

To enhance the performance of the C_2_N device displayed in Fig. 1, the C_2_N electrodes were replaced with gold electrodes and a gate terminal was placed underneath the channel producing a C_2_N based field effect transistor displayed in Fig. 4 for the purpose of detecting propane, butane gas molecules. The gate terminal consists of dual layers: a dielectric layer composed of SiO_2_ with a dielectric constant of 3.9 and a metallic layer of 2.9 Å. The best results were obtained when the gate voltage was fixed at − 5 V and the bias voltage was fixed at 0.4 V.

For the purpose of analyzing the electronic transport properties, several factors such as the device's electronic current, density of states, and transmission spectrum were investigated for both the bare C_2_N device displayed in Fig. 1 and the C_2_N transistor displayed in Fig. 4 with each of the target gas molecules.

### Computational method

The investigation of this sensor employed first-principles modeling techniques for accurate simulations. The geometry optimization of the sensor was performed using the Generalized Gradient Approximation (GGA) method in conjunction with density functional theory (DFT). The relaxation of the atomic structure continued until the maximum atomic force reached below 0.05 eV/Å. Optimization was performed individually for each of the gas molecules. Furthermore, the C_2_N atoms underwent optimization before forming the sensor. Finally, the complete sensor, including each target molecule, was optimized as a whole. The optimization process employed a 1 × 1 × 1 k-mesh and the Monkhorst–Pack grid, which is a well-established uniform grid known for its reliable convergence, as demonstrated in previous studies^38^. The mesh cut-off energy was set at 80 Ha.

In order to precisely capture the electronic transport properties, a more extensive k-mesh grid was utilized. Recommendations from the Quantumatk website^39^ and other sources^40^ propose the usage of 100 k-points in the transport direction, specifically the C direction illustrated in Fig. 1. Previous investigations carried out by Thomas et al.^41^ employed a 1 × 1 × 100 k-point sampling approach along the device transport direction for IV calculations. In this research, a 5 × 5 × 100 k-point sampling scheme was employed to discretize the Brillouin zone, ensuring accurate outcomes.

To study of the electronic properties, non-equilibrium Green's function (NEGF) combined with DFT were employed. The positioning of gas molecules on the C_2_N monolayer were used to analyze the transport properties of both the C_2_N monolayer and the sensor with each target molecule. The system consists of three regions: the scattering region containing each gas molecule, the right electrode, and the left electrode. The DFT approach was used to measure the transverse current, transmission spectrum and, the projected device density of states. The grid mesh cut-off energy used for these calculations was set at 80 Hartree. The ATK-VNL calculations and equations employed in this study are elaborated and presented in previous research work^20, 33^.

## Results and discussion

This section presents and discusses the detailed results generated from ATK-VNL, focusing on the calculated current–voltage (IV), and density of states (DOS) properties of the simulated C_2_N-based sensor. The objective is to investigate the practicality of the designed C_2_N sensor in detecting specific target molecules. The transport characteristics were analyzed for the C_2_N system, including the transmission spectrum, density of states, current–voltage characteristics, and current variations. These properties were evaluated for various scenarios, namely the C_2_N sensor alone, the C_2_N system with the addition of a propane molecule, the C_2_N sensor with the addition of a butane molecule, and the C_2_N system with the addition of both propane and butane molecules. Furthermore, the impact of higher concentrations of propane and butane gases on the sensor's performance was also investigated.

The binding energy is a crucial parameter in understanding the interaction between gas molecules and the sensor surface, exhibits distinct values for different gases. In this work, the binding energy is quantified as − 0.82 eV for butane, − 0.39 eV for propane, and notably, − 4 eV for both gas molecules. This metric serves as an indicator of the strength of the interaction between the gas molecules and the sensor surface. Specifically, a higher binding energy, as observed in the case of both butane and propane, signifies a robust and stable interaction due to the formation of strong chemical bonds, which can lead to heightened sensitivity and selectivity in gas detection. Conversely, the lower binding energy for propane, while still indicative of a significant interaction, may suggest a somewhat weaker attachment.

The simulation consistently yields identical results, demonstrating the reproducibility of the data. In contrast, during experimental testing, a distinct protocol is employed to maintain data integrity. Following each test, the sensor undergoes a thorough cleaning process involving the use of distilled water, ensuring that any residual effects from previous tests are mitigated. Subsequently, the sensor is diligently dried before the next testing cycle commences. This thorough cleaning and preparation regimen helps maintain the sensor's sensitivity and reliability, ensuring that each experimental data point is taken under consistent and controlled conditions.

### Device density of states (DDOS)

**Figure 5:**
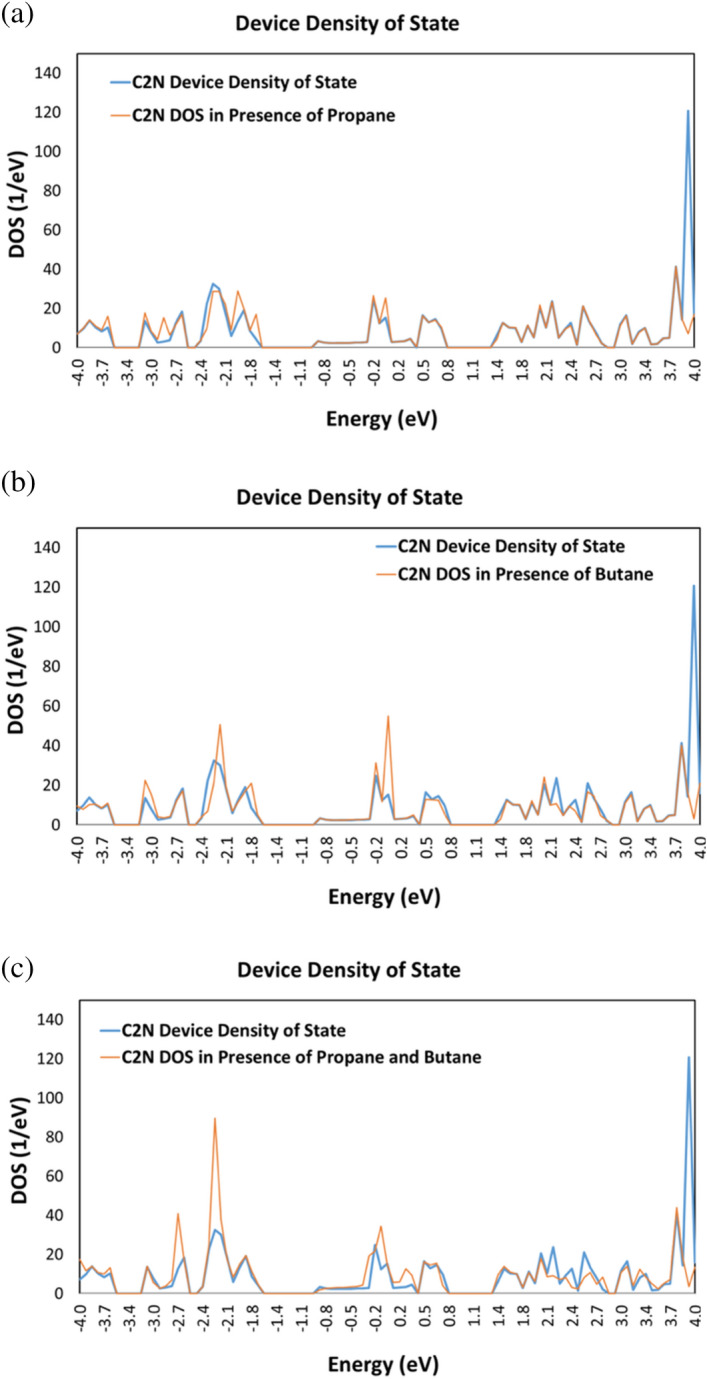
The device density of states (DOS) of the simulated C_2_N sensor undergoes alterations when exposed to different molecules, namely (**a**) propane, (**b**) butane, and (c) propane and butane.

The identification of butane and propane gases is evident by the distinct and observable alterations in the C_2_N Device DOS. Figure 5 presents a comparison of the DDOS between the C_2_N -sensor with and without each gas molecule. It is apparent from Fig. 5 that the C_2_N sensor without a gas molecule has different number of energy states compared to the C_2_N sensor with the addition of each target molecule. The introduction of propane initiates a variation in the energy spike at − 3.7, − 2.8, − 2.3, − 2, − 1.9, − 1.8, 0, and 3.9 eV as displayed in Fig. 5a. On the other hand, the addition of butane leads to higher energy spikes at the energy levels − 3.8, − 3.1, − 2.2, − 1.9, − 0.2, and 0 eV, and lower energy spikes appear at the energy levels 2.1, 2.4, and 3.9 eV as shown in Fig. 5b. Similarly, when exposed to both propane and butane molecules, the C_2_N sensor exhibits a significant shift in DDOS, as illustrated in Fig. 5c where higher energy spikes appear at − 2.7, − 2.3, − 0.2, and 0.2 eV while lower energy spikes are at 2.1, 2.3, 2.5, and 3.9 eV.

**Figure 6:**
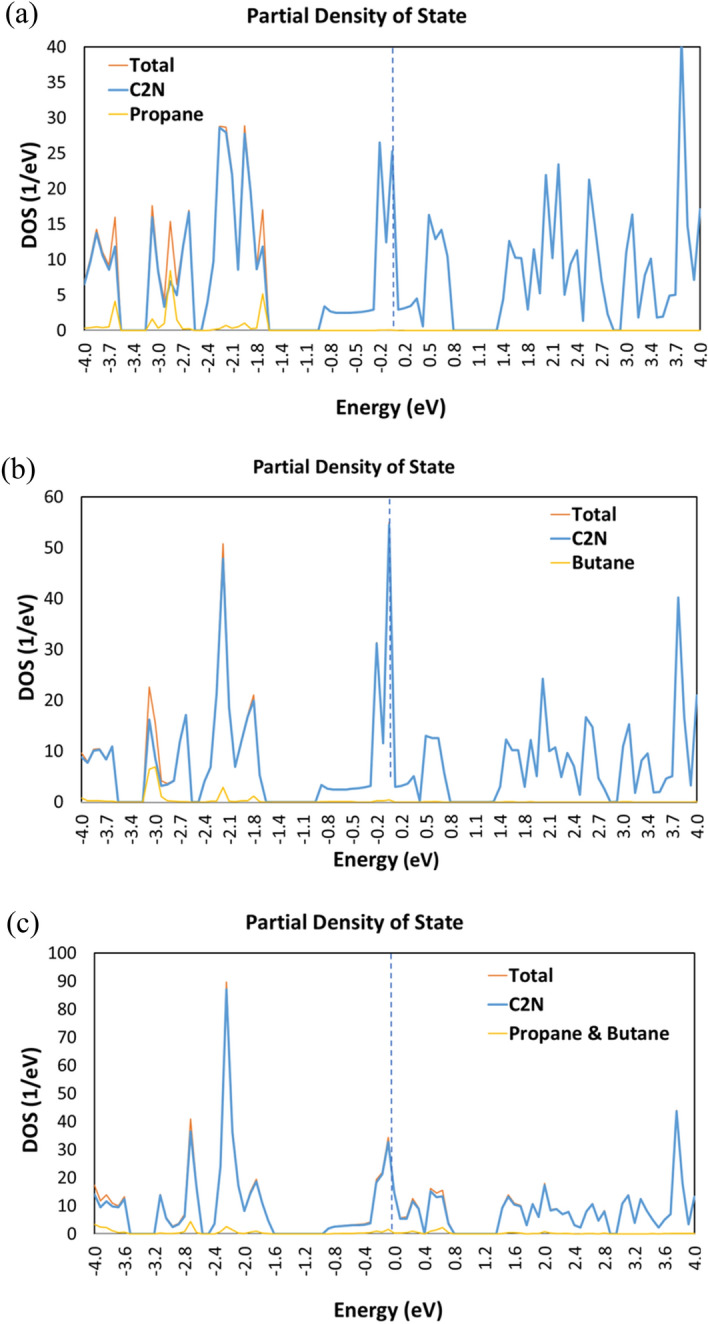
Total and partial density of states (DOS) of C_2_N sensor in the addition of (**a**) Propane molecule; (**b**) Butane molecule; and (**c**) Propane and Butane molecules.

Figure 6 provides a comprehensive analysis of the partial density of states (DOS), illustrating the impact of detecting propane and butane molecules on the DDOS. Figure 6 shows that when these target molecules are introduced to the C_2_N device, a observable peak in the DDOS can be noticed for each target molecule. The appearance of this peak reflects a distinctive interaction between the propane and butane molecules and the C_2_N channel, leading to the creation of new electronic states within the relevant energy range. This interaction induces changes in the electronic structure of the channel, thereby causing alterations in the DDOS. The variations in the DDOS can be justified by various factors, such as the transfer of electrons between the channel material and the propane and butane molecules, as well as the formation of chemical bonds among the C_2_N channel and the target molecules.

One possible justification for the variations in the electronic structure of the material when identifying propane and butane is that the addition of these molecules introduces defects or impurities, thereby influencing the material's electronic characteristics. These variations become evident as extra energy levels appearing in the DOS, consequently affecting its density. Moreover, these impurities can affect the symmetry of electronic states, leading to variations in the DOS. Additionally, the chemical and mechanical properties of the material may also undergo transformations, leading to modifications in the DOS. The extent of these variations relies on the type and concentration of the detected propane and butane molecules.

### Transmission spectrum

**Figure 7:**
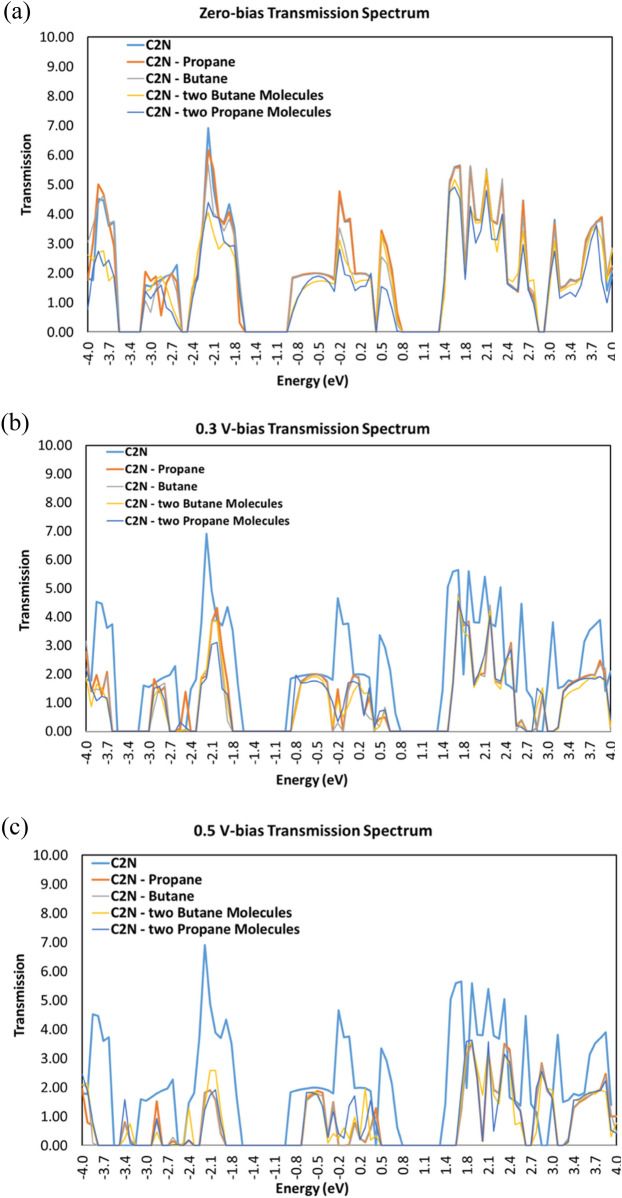
Transmission spectra T(E) for C_2_N sensor in the addition of propane molecule, butane molecule, two butane molecules, and two propane molecules (**a**) bias voltage = 0 V, (**b**) bias voltage = 0.3 V, and (**c**) bias voltage = 0.5 V.

Figure 7 displays the transmission spectra T(E) for the C_2_N sensor with and without each of the gas molecules (propane, and butane) at various voltages: (a) bias voltage = 0 V, (b) bias voltage = 0.3 V, and bias voltage = 0.5 V. The figure displays the modifications in transmission signal when different gas molecules are placed on the sensor channel at various voltages.

### Current–voltage

**Figure 8:**
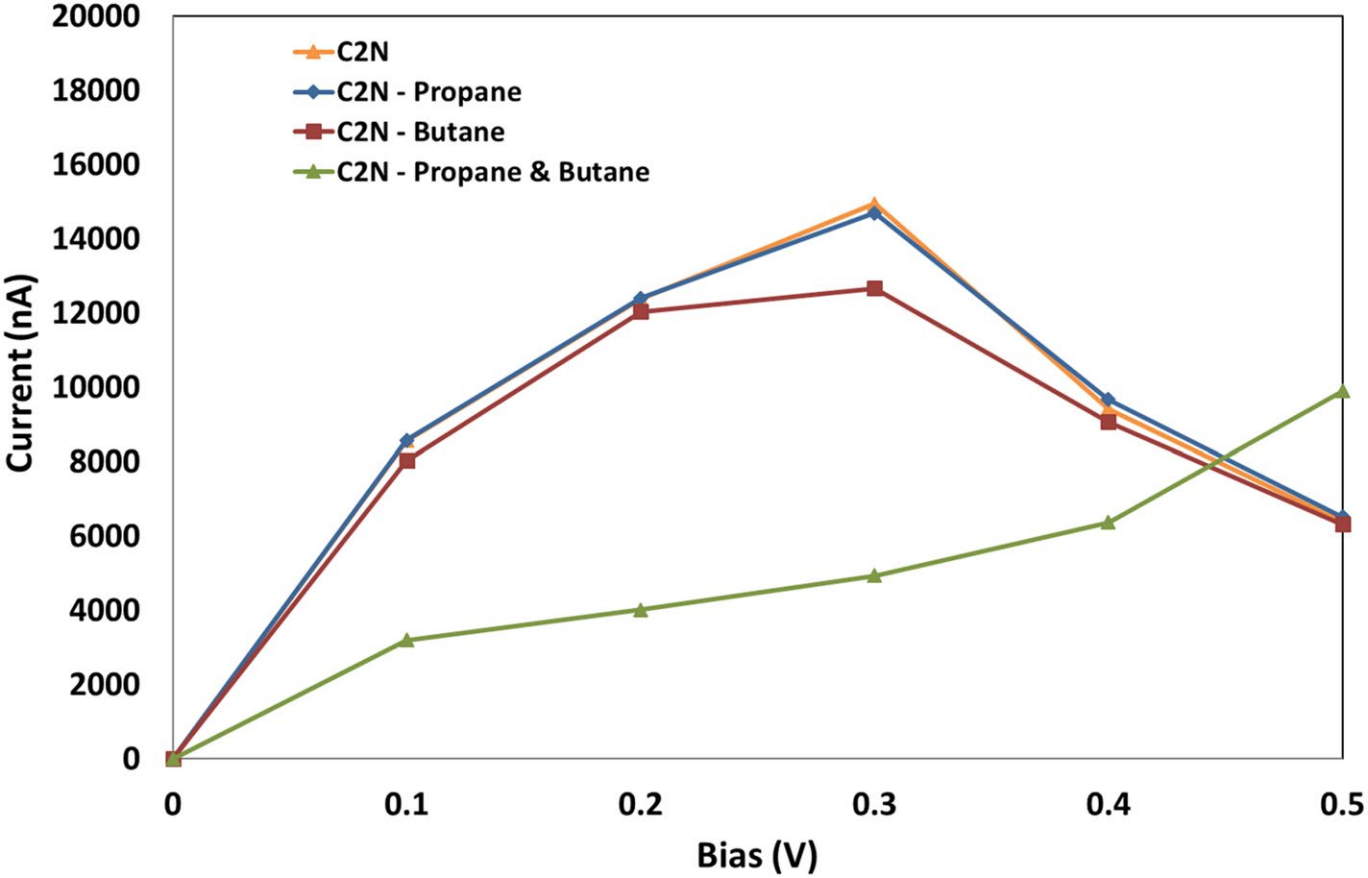
Current–voltage characteristics vs bias for the C_2_N sensor (orange), for the C_2_N sensor with propane (blue), for the C_2_N sensor with butane (red), and for the C_2_N sensor with both propane and butane.

The current behavior of the C_2_N sensor exhibits distinct variations when detecting propane and butane molecules, as demonstrated in Fig. 8. Each propane and butane molecule interacts with the C_2_N sensor channel in a unique manner, influencing its size, state, and mode of interaction.

**Figure 9:**
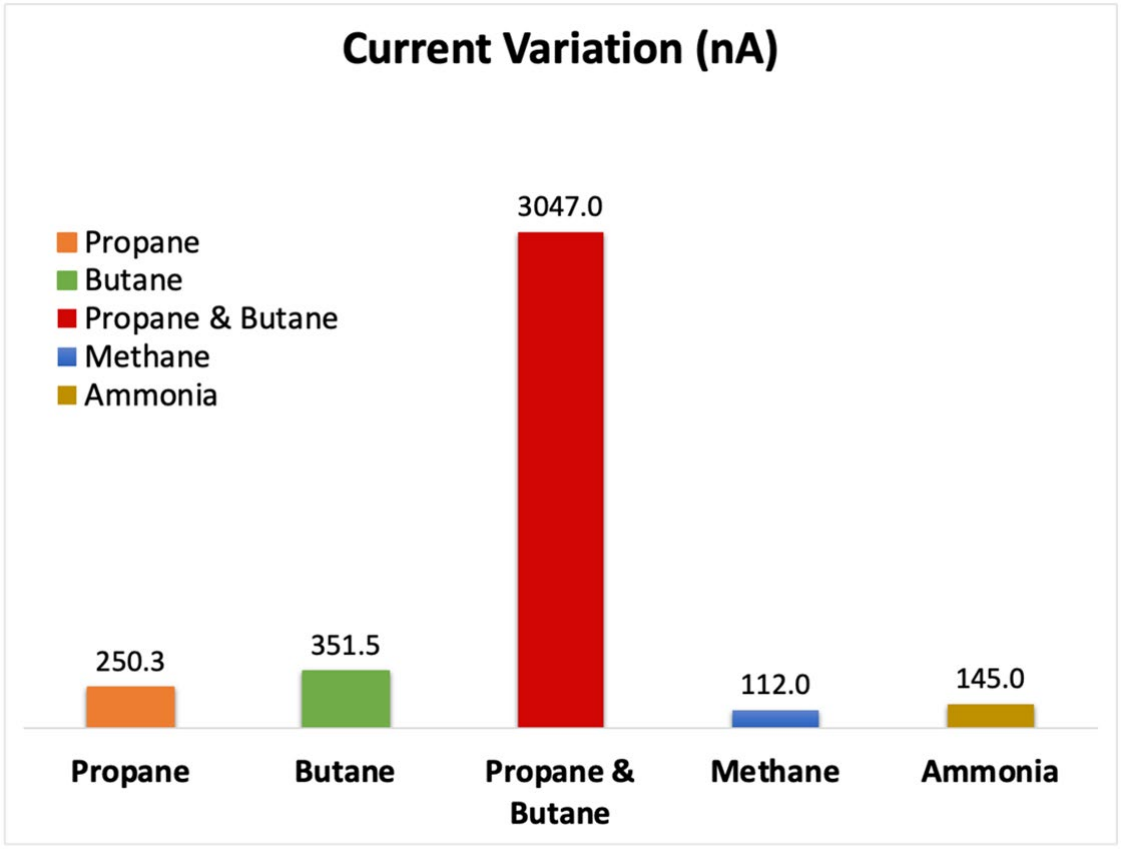
Variation in C_2_N sensor electrical drain current in the addition of propane molecule, butane molecule, both of propane and butane molecules. Mehtane molecule, and Ammonia molecule.

**Figure 10:**
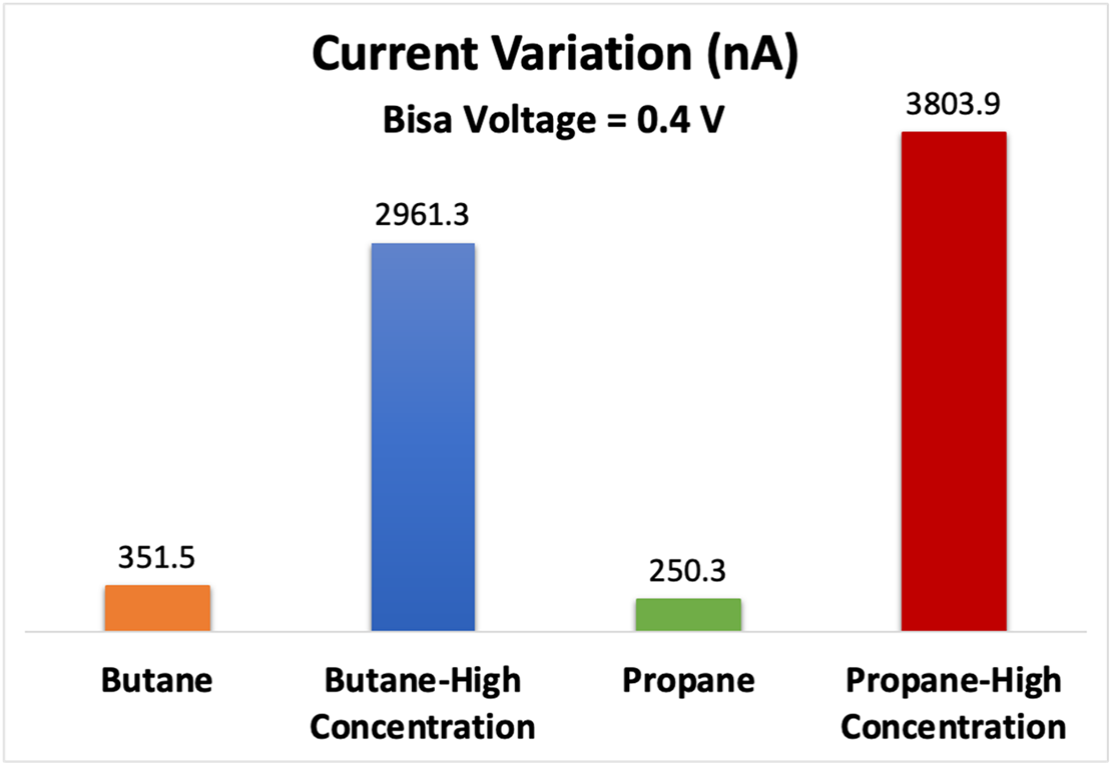
Variation in C_2_N sensor electrical drain current in the addition of butane molecule, two butane molecules (higher concentration), propane molecule, and two propane molecules (higher concentration).

Figure 9 illustrates the response of the designed system in terms of the variation in current, with the most significant change in the electrical signal observed during the adsorption of both propane and butane molecules. These findings indicate that the sensor exhibits high sensitivity for propane and butane, resulting in a unique electrical current for each molecule. The variation in current can be attributed to changes in charge and electrical potential upon the introduction of the target molecules, which subsequently alters the density of charge carriers. As a result, the sensor's conductivity and current undergo changes. The selectivity of the C_2_N sensor was tested as displayed in Fig. 9, revealing the highest selectivity for butane and propane gases, with a current variation of 250 and 350, respectively. This heightened response to butane and propane shows its efficacy in detecting these gases. Additionally, the sensor exhibited low variations in current when exposed to methane and ammonia, registering at 112 and 145 nA, respectively. This confirms the sensor's selectivity toward butane and propane molecules. Figure 10 illustrates the changes in current (at V_ds_ = 0.4 V) upon the introduction of varying concentrations of propane or butane molecules. The figure demonstrates a direct correlation among the concentration of the target molecules and the magnitude of the current variation. Specifically, higher concentrations of the target molecules lead to larger changes in the electrical current readings. This observation suggests a stronger adsorption effect due to the higher concentration. Importantly, our findings align with previous research in this field^4^, supporting the validity of our results.

**Figure 11:**
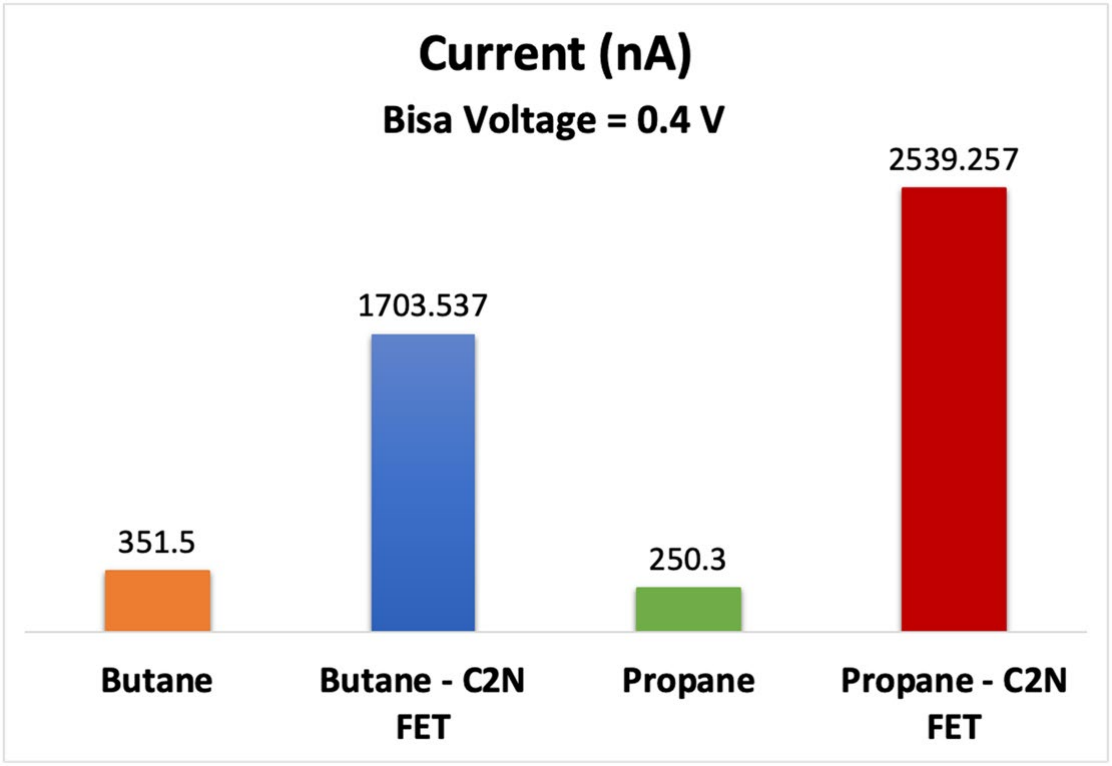
Variation in electrical drain current for C_2_N sensor in comparison with the C_2_N FET due to the addition of propane gas molecule and butane gas molecule (Gate voltage = − 5 V).

In order to improve the performance of the C_2_N device displayed in Fig. 1, several modifications were made. Firstly, the C_2_N electrodes were replaced with gold electrodes, and a gate was incorporated beneath the channel. Subsequently, the target molecules were introduced to the channel, and the resulting current variation was measured. The simulation results, as depicted in Fig. 11, indicated that the new sensor exhibited improved performance and higher sensitivity compared to the previous configuration. By maintaining a bias voltage of 0.4 V across the right and left electrodes, the C_2_N FET sensor achieved optimal performance. The sensitivity of the sensor was particularly notable when the bias voltage was set to 0.4 V, as depicted in the Fig. 11. This work is a proof of concept, showcasing the capability of the developed C_2_N device to effectively detect propane and butane molecules.

The addition of the gate terminal contributed to the enhanced sensor performance. The gate serves as an additional control element that enables better manipulation of the electronic properties of the channel. By applying a voltage to the gate, the electrostatic environment near the channel can be effectively modified. This enables more precise control over the sensing process and facilitates a higher sensitivity to the target molecules.

Overall, the integration of gold electrodes and the addition of a gate terminal played crucial roles in improving the C_2_N sensor's performance, ultimately leading to enhanced sensitivity and more accurate detection capabilities.

In previous research^4^, Graphene Nanoribbon Field Transistors were used for the detection of propane and butane gases. However, our study reveals a noteworthy improvement in the detection performance when employing C_2_N material. This enhanced performance can be attributed to several key factors. First, C_2_N exhibits an elevated sensitivity to the presence of propane and butane molecules, allowing for the detection of even trace amounts of these gases. Second, its unique molecular structure facilitates stronger and more specific interactions with propane and butane molecules, resulting in a more accurate and reliable detection mechanism. Furthermore, the properties of C_2_N material can be fine-tuned to optimize its performance for the detection of these specific gases, making it a versatile and adaptable choice for gas sensing applications. Additionally, C_2_N-based sensors typically exhibit lower noise levels, leading to more precise measurements and a higher signal-to-noise ratio. Finally, C_2_N material's improved selectivity in differentiating between gases and potential interference reduces the likelihood of false alarms or inaccuracies. Overall, the utilization of C_2_N material in our research offers a substantial advantage over Graphene Nanoribbon Field Transistors, making it a promising candidate for the detection of propane and butane gases.

This study serves as a proof of concept for the practical application of C_2_N transistors in gas detection. The C_2_N transistor's remarkable capability is demonstrated in its capacity to register substantial variations in current upon exposure to gas molecules, with the detection signal intensifying proportionally as gas concentrations increase. The fabrication of this highly promising sensor requires a structured process, designed to optimize its performance.

The process initiates with a precise material preparation stage, whereby C_2_N sheets are synthesized utilizing chemical vapor deposition (CVD) or other suitable methods. Subsequent to material synthesis, the deposition of gold electrodes onto the C_2_N sheets is carried out with utmost precision using physical vapor deposition (PVD) or sputtering techniques. These gold electrodes function as vital contact points essential for subsequent electrical measurements.

The sensor structure is configured through the application of photolithography, allowing for the precise delineation of the sensor's geometry, including the positioning of electrodes and the channel. The next crucial step in this process involves the utilization of reactive ion etching (RIE) or comparable etching methodologies to create the desired patterns within the C_2_N sheet. This results in the creation of a C_2_N channel that effectively connects the gold electrodes.

The integration of a gate terminal beneath the C2N channel is conducted with precision. This is accomplished by depositing a suitable gate material, such as silicon dioxide, and subsequently applying a dielectric layer. This dielectric layer serves the vital function of insulating the gate terminal from the channel, consequently amplifying the sensor's overall performance and sensitivity. This suggested fabrication process underscores the potential of the C_2_N-based gas sensor in delivering accurate and highly sensitive gas detection capabilities, offering significant promise for a range of critical applications.

## Conclusion

The objective of this article is to make a significant contribution to the existing field by designing a new and affordable two-dimensional nitrogenated holey graphene nanoribbon sensor for detecting gas leaks in households. The simulations for the gas sensors of butane and propane are carried out using the QuantumWise software. The calculated and discussed factors consist of the device density of states (DDOS) and the transmission spectrum of the device in close proximity to gas molecules. In order to detect gas molecules, the simulated sensor operates by monitoring any changes in electric current passing across the device. In conclusion, this study introduces the use of C_2_N sensors for the detection of butane and propane gases. By replacing the electrodes with gold electrodes and incorporating a gate terminal, the sensor's performance was greatly enhanced, resulting in improved sensitivity and precise detection capabilities. The findings highlight the potential of C_2_N sensors as cost-effective and efficient solutions for gas sensing applications. The ability to detect propane and butane gases, which are commonly used fuels, holds significant importance in preventing leakage and explosion accidents in household and industrial settings. By leveraging the unique properties of C_2_N and employing advanced simulation tools, this study contributes to the advancement of gas sensing technology.

## Data Availability

All data generated or analyzed during this study are included in this published article.
